# Building the evidence base to optimize the impact of key population programming across the HIV cascade

**DOI:** 10.1002/jia2.25146

**Published:** 2018-07-22

**Authors:** R Cameron Wolf, Trista Bingham, Greg Millett, Rose Wilcher

**Affiliations:** ^1^ U.S. Agency for International Development Office of HIV/AIDS Washington DC USA; ^2^ U.S. Centers for Disease Control and Prevention Atlanta GA USA; ^3^ AmfAR The Foundation for AIDS Research New York NY USA; ^4^ FHI 360 LINKAGES Project Durham NC USA

**Keywords:** key populations, men who have sex with men, sex workers, people who inject drugs, transgender persons, HIV, service delivery, implementation science

## 1 Global context

The most recent global HIV data have brought great optimism that controlling the HIV epidemic could become a reality. These encouraging data show overall declines in both AIDS‐related deaths and new HIV infections worldwide 1. Recent data also demonstrate impressive gains toward the global 90‐90‐90 targets. As of 2016, an estimated 70% of all people living with HIV (PLHIV) globally knew their HIV status. Among those who had been diagnosed, 77% were accessing antiretroviral therapy, and 82% of people on treatment had achieved viral suppression 1.

Despite this progress, the optimism is tempered by concern that reducing HIV incidence rates must be further accelerated to guarantee epidemic control 2. Moreover, the recent gains have not been uniform. While global data indicate important achievements in addressing the epidemic among key populations – defined by the World Health Organization (WHO) as men who have sex with men (MSM), sex workers, transgender people, people who inject drugs (PWID), and prisoners 3 – these gains still lag far behind those made in the general population.

UNAIDS estimates that 44% of all new HIV infections among adults worldwide occur among key populations and their partners 1. In generalized epidemic contexts of sub‐Saharan Africa, key populations and their sexual partners account for 25% of new HIV infections, while in concentrated epidemic settings, they account for as much as 80% of infections 1. Globally, sex workers, MSM and PWID are 10, 24 and 24 times more likely, respectively, to acquire HIV compared with the general population ages 15 years and older 4. Transgender women are 49 times more likely to be living with HIV and prisoners are five times more likely to be living with HIV compared to other adults 4, 5.

## 2 Growth in supporting HIV programmes for key populations

Evidence of the disproportionate epidemiological burden that members of key populations shoulder has been met with important policy developments and funding commitments. In 2014, the Global Fund to Fight AIDS, Tuberculosis and Malaria launched the Key Populations Action Plan, reflecting its commitment to help meet their HIV prevention, care and treatment needs and rights 6. That same year, WHO released consolidated guidelines on HIV prevention, diagnosis, treatment and care for key populations 3. These guidelines were updated in 2016 to reflect the urgent call to treat all individuals regardless of CD4 count and to provide pre‐exposure prophylaxis (PrEP) to those “at substantial risk” 7. Additional global implementation guidance and programmatic tools soon followed to support key population programme design and scale up 8, 9, 10, 11, 12.

The President's Emergency Plan for AIDS Relief (PEPFAR) has also launched specific initiatives to expand key populations’ access to and retention in HIV services. Through programmes such as the Key Populations Challenge Fund, the Key Populations Implementation Science Initiative and the Local Capacity Initiative, PEPFAR supported work to understand and better serve key populations, as well as to strengthen capacity of key population‐led organizations to address the epidemic in their communities 13. Moreover, PEPFAR recognizes that “ensuring key populations have access to and increase their use of comprehensive packages of health and social services” is essential for achieving epidemic control 14.

Finally, the UNAIDS HIV Prevention 2020 Roadmap deems combination prevention programmes for key populations necessary to accelerate declines in new HIV infections at country level. The Roadmap calls for combination prevention programmes that are evidence‐informed, community‐owned, and human rights‐based; implemented at scale; and tailored to the specific needs of key populations 15.

## 3 Key populations, data challenges and the HIV cascade

These commitments are critical to advancing a more effective response. However, progress translating commitments into improved outcomes for key populations has been hindered by persistent barriers, such as stigma (including self‐stigma), discrimination, and punitive legal and policy environments. In addition, the field faces ever‐present data challenges with key populations, who often do not self‐disclose their current or former status as key population members. Consequently, they may be included as members of the “general population” and their contribution to HIV transmission underreported and unrecognized. Alternatively, they may be connected to key population community services in one place but receive testing or treatment services anonymously in another, making it difficult for programmes to track and support clients across multiple service points.

The limited population‐based data that are available show that testing and treatment coverage among key populations remains disproportionately low with no key population group close to achieving 90‐90‐90 targets 1, 16. This has led to calls for improving outcomes for key populations through data‐driven interventions. Indeed, this supplement grew out of the urgent need to share the emerging evidence from both new and evolving service delivery interventions for key populations.

**Figure 1:**
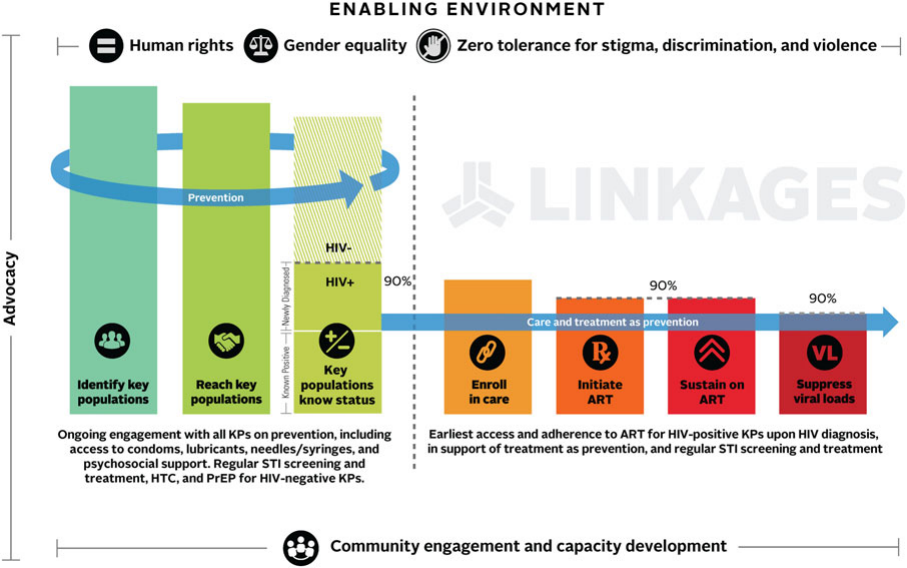
Cascade of HIV prevention, care, and treatment services for key populations

The HIV prevention, care and treatment cascade has been globally adopted as a useful framework for guiding key population programming (Figure 1) 8. It can indicate where programmatic efforts are falling short in reaching and retaining key populations across the continuum of care, and thereby pinpoint areas for intensified work. Moreover, this cascade model highlights the importance of engaging and building capacity of communities to lead efforts to reach, test, treat, and retain key populations in services, as well as the need to tackle structural barriers – including stigma, discrimination, violence, gender‐based bias and, in many cases, criminalization. Cutting across the cascade is the need to ensure programmatic efforts are rights based and that confidentiality, safety and security are respected.

Achieving epidemic control will not be possible without more robust and rapid progress in delivering evidence‐based interventions that improve key populations’ access to and uptake of HIV services across the cascade. Fundamental to that progress is the generation and use of key population‐specific cascade data. The recently relaunched Key Populations Atlas from UNAIDS represents an important step in that direction 17. This tool brings together country‐specific data on a variety of indicators disaggregated by key population group. We need to complement this with better data from a variety of methodological approaches that identify strategies effective at reaching and engaging key populations at different points along the HIV cascade, and allow targeted investments in programming at those points where they are most needed.

## 4 Towards a more effective response for key populations

Recognition of these needs has led to advances in monitoring key populations’ uptake of services across the cascade to identify “leaks” in the system, as well as more sophisticated analysis and use of data to identify solutions and strengthen programming 18, 19. In addition, a number of key population‐focused implementation science studies are underway across a range of geographies to evaluate the effectiveness of new approaches, outreach strategies and delivery modalities in overcoming structural obstacles and improving service uptake and retention with different key population groups 20.

As programming is scaled up globally, it is critical that we maximize public health impact by sharing the latest evidence of what works to engage key populations in targeted prevention, treatment, and retention programmes. The contents of this supplement represent high‐quality articles from a range of multidisciplinary efforts to advance key population science and practice across the cascade. They offer new evidence and data‐driven strategies for improving programming with MSM, sex workers, transgender people and PWID across diverse geographies. The supplement does not contain articles addressing prisoners as they require significantly different approaches from key populations in communities outside incarcerated settings.

## 5 Data approaches to improve cascade monitoring

Five of the papers in this supplement describe methods and analyses specific to key populations that can be used to refine and focus interventions. The data generated from these approaches are important to guide strategic planning, resource allocation and programme quality improvement initiatives.

The supplement opens with a commentary by Hakim *et al*. in which the authors make the case for why we need better key population cascade data and how we can get it 21. They argue that targeted bio‐behavioural surveys represent an important source of data to guide the epidemic response but have been underutilised to monitor and inform key population service delivery efforts. While there may be sampling concerns and other limitations to these types of surveys, the authors underscore that bio‐behavioural survey data are critical to triangulate with available programme data for a comprehensive assessment of the reach and impact of services for key populations.

An article by Mukandavire *et al*. presents a new methodology to estimate the contribution of onward HIV transmission among key populations to the overall HIV dynamic in Dakar, Senegal 22. They report that the contribution of commercial sex to HIV transmission is diminishing; however, unprotected sex between men contributed to 42% of transmissions between 1995 and 2005, and increases to an estimated 64% in the 2015 to 2025 period. The authors posit that this dynamic may also be observed in other low‐ and middle‐income countries where the contribution of MSM to overall HIV transmission may be under‐appreciated.

To better refine key population programming at country level, Lillie *et al*. describe a partnership between PEPFAR and The Global Fund to conduct key populations cascade assessments 23. By jointly participating in these assessments, major funders and national stakeholders are able to better align packages of services, training, geographic coverage, innovations, data collection and quality improvement efforts. These cascade assessments were completed in eight countries: Malawi, Cameroon, Swaziland, Haiti, Angola, Nepal, Cote d'Ivoire and Botswana. For this commentary, the authors review common challenges and recommendations made at the programme, national and donor level at each step in the cascade.

Using data collected from an online survey implemented through the gay social networking application, Hornet, Ayala *et al*. describe determinants of HIV service uptake among a global sample of MSM 24. Of the 10,774 HIV‐negative respondents, 13% reported PrEP use. Among HIV‐positive respondents (n = 1243), both ART use and undetectable viral load (UVL) were associated with older age, a recent sexually transmitted infection (STI) test or STI treatment; and awareness of unlikely HIV transmission with UVL. The findings underscore the importance of STI testing and treatment as well as information about HIV transmissibility (U = U) for encouraging PrEP and ART use. This study is noteworthy for its innovative use of a gay dating app to rapidly generate data from a large online community of MSM that can be used for advocacy and tailored programme decision‐making.

Finally, Suraratcheda *et al*. contribute the first estimates on the costs and cost‐effectiveness of providing oral PrEP for MSM in Thailand 25. Costing studies related to key population programming are extremely limited yet costing data are critical for effective programme planning. This paper makes an important first contribution for the Asia‐Pacific region by estimating the annual costs (US$223 to US$331 per MSM per year, including demand creation activities) and cost‐effectiveness of PrEP under several delivery scenarios. While providing PrEP to all MSM over the next five years would have greater epidemiological and economic benefit to Thailand, the authors conclude that providing PrEP to high‐risk MSM would be the most cost‐effective approach.

## 6 Improving recruitment, testing uptake and case finding

The supplement features two articles that broaden our understanding of strategies for improving the reach of prevention services and uptake of HIV testing among previously unreached key population members. Herce *et al*. report data from two bio‐behavioural surveys in Malawi and Angola that illustrate the value of providing venue‐based outreach and testing services in “hotspots,” where people including MSM, FSWs and transgender people meet and seek sex partners 26. Over 70% of the individuals diagnosed with HIV through the venue‐based approach were not previously aware of their status, indicating that this was effective at increasing testing uptake and case finding among these populations.

A study by Kan *et al*. from Tajikistan compares the effectiveness of three network‐based approaches to recruitment and case finding among PWID 27. The approaches include two respondent‐driven sampling (RDS) strategies – one restricted and the other unrestricted – and an active case‐finding (ACF) strategy that involves direct outreach by peers who are living with HIV or current/former PWID. Collectively, these approaches identified 190 new cases of HIV in an eight‐month period, linked 80% of them to confirmatory testing, and initiated 87.5% of the confirmed positives on treatment. While RDS strategies were more effective than ACF in detecting new HIV cases, the ACF approach attracted a higher proportion of first‐time testers. This finding led the authors to note that both strategies are likely needed to achieve their case‐finding goals among PWID in this setting.

## 7 Innovations in HIV testing modalities and linkage to treatment

Recognizing that innovations in HIV testing options are needed to improve coverage, three articles in the supplement examine feasibility, acceptability, and effectiveness of new HIV testing modalities. Tun *et al*. present the results of a pilot intervention to distribute oral HIV self‐testing kits to MSM through key opinion leaders in Lagos, Nigeria 28. This study found that not only is oral self‐testing feasible and highly acceptable among MSM in this urban population, but also that effective linkage to treatment can be achieved for those who test positive through self‐testing with active follow‐up and access to a trusted MSM‐friendly community clinic that offers HIV treatment.

In a study from Vietnam, Green *et al*. explore HIV testing interventions through MSM lay providers and HIV self‐testing, promoted through online channels and face‐to‐face interactions 29. The study found that more than half of the MSM who sought lay‐ or self‐testing were first‐time testers. These new testing strategies resulted in higher detection of new HIV cases (6.8%) compared to conventional facility‐based testing (estimated at 1.6%), while those linked to testing from social media interventions presented with even higher HIV‐positive people (11.6%). Moreover, 90% of those identified as positive were successfully registered for ART.

A study from Thailand also demonstrates the promise of leveraging technology and self‐testing to improve reach and testing uptake among MSM and transgender women 30. In this study, Phanuphak *et al*. explore preferences among three different modalities of HIV testing including: (1) offline HIV counselling and testing; (2) online pre‐test counselling and offline HIV testing; and (3) online counselling and online, supervised, HIV self‐testing. The study demonstrated that online counselling coupled with online, supervised, HIV self‐testing is feasible and acceptable. In addition, the online strategy produced the highest proportion of first time testers (47.3%) and had the highest HIV prevalence (15.9%). Being a transgender woman and spending more than four hours per day on social media increased a participant's likelihood to self‐select for online counselling and HIV testing.

## 8 Taking community‐led programming across the continuum to scale

Two papers in the supplement report outcomes from large‐scale, key population‐led efforts to improve outcomes across the cascade. Ndori‐Mharadze *et al*. report results from an evaluation of ‘Sisters with a Voice’, Zimbabwe's nationally scaled comprehensive programme for FSWs, following intensified community mobilization activities 31. The findings demonstrate that early peer mobilization efforts to familiarize community members with tailored HIV services were associated with improved outcomes, notably increases in HIV testing frequency, knowledge of HIV status and increased linkage to ART.

Results of an innovative HIV self‐testing component within a broader, community‐wide implementation science project in Curitiba, Brazil demonstrated feasibility and improved HIV diagnosis among young MSM who had not previously tested for HIV 32. Based on their findings, De Boni *et al*. report on the expansion and tailoring of the Internet‐based self‐testing platform to increase HIV testing coverage among MSM in São Paulo, Brazil's largest metropolitan area with the highest number of new HIV infections.

## 9 Addressing structural barriers

Due to the criminalized and stigmatized nature of key populations globally, sex workers, MSM, PWID and transgender people are often afraid to visit healthcare services and, when they do go, are reluctant to disclose their sexual histories for fear of rejection, derision or other negative reactions from providers 33, 34. In addition, the perpetration of violence against key populations is frequent and often severe. Experiences of violence not only increase the risk of key populations acquiring HIV but also deeply affect their desire and ability to get tested for HIV and adhere to HIV treatment 35, 36.

Two papers in this supplement address structural barriers to better HIV‐related outcomes. Bhattacharjee *et al*. describe successful efforts to integrate violence prevention and response services into the national key population programme in Kenya 37. Drawing on programme data over a four‐year period, this paper contributes important evidence that it is possible to address violence against key populations under the leadership of the national government, even in an environment where sex work, same‐sex sexual practices and drug use are criminalized 38.

A commentary by Friedland *et al*. reflects on the evolution of the PLHIV Stigma Index, which at the end of 2017 had interviewed more than 100,000 PLHIV in 90 countries 39. The paper describes efforts at updating the new PLHIV Stigma Index 2.0 to better capture HIV‐related *and key population‐related* stigma, within the context of modern global testing and treatment guidelines. The updated tool was pilot‐tested through a community‐led process in Cameroon, Senegal and Uganda, and provides essential evidence and opportunities for communities in other countries to more effectively document stigma and advocate for and implement stigma mitigation interventions as part of human rights‐affirming HIV responses.

## 10 Moving ahead

These papers provide much‐needed contributions to the evidence base for key population programming across the HIV prevention, care and treatment cascade. Our hope is that this supplement will compel funders, policymakers, implementers and other stakeholders to do more now to champion data‐driven programming. One common theme that emerges from this supplement is that we should establish and scale‐up innovative, community‐led services, while expanding the integration and options for key populations within the health system. In addition, we will not make sustainable improvements in outcomes if we do not better address the stigma, discrimination and violence that key populations experience at the hands of family, community members, health care providers and the state.

As more evidence on key population programmes emerges, it is critical that the international community catalyse these advances with supportive policies that promote widespread uptake of effective approaches. Important studies are ongoing through the PEPFAR Key Populations Implementation Science and amfAR Implementation Science Grants initiatives, from which we anticipate more rich data designed to fill further gaps in our understanding of how to implement better services for key populations.

After more than three decades in the fight against HIV, plans to end the HIV epidemic through goals such as UNAIDS 90‐90‐90 have been adopted by governments, major donors, and stakeholders globally. Investments to address the epidemic among key populations should be central to these efforts. With ever‐present threats of stigma, discrimination, violence, and other human rights abuses, the gains that have been made among key populations are precarious. The urgency of continuing to maintain focus on these groups cannot be understated. To leave no one behind, the substantial progress that has been made to date against the epidemic will need to be bolstered with rigorous, key population‐specific data collection and use, with partnerships focused on vigilance, courage, tolerance and commitment.

## Competing interests

The authors have no other funding or conflicts of interest to disclose.

## Authors’ contributions

RCW, TB, GM and RW all contributed to the preparation of the first draft. All authors approved the final manuscript.

## Funding

This manuscript was supported by multiple agencies including the United States Agency for International Development (USAID) and the U.S. President's Emergency Plan for AIDS Relief (PEPFAR) through the Linkages across the Continuum of HIV Services for Key Populations Affected by HIV project (LINKAGES, Cooperative Agreement AID‐OAA‐A‐14‐00,045); PEPFAR through the Centers for Disease Control and Prevention (CDC); and amfAR, the Foundation for AIDS Research. The content is solely the responsibility of the authors and does not necessarily represent the official views of any of the funding agencies.
